# Assessment of fluid responsiveness by inferior vena cava diameter variation in post‐pneumonectomy patients

**DOI:** 10.1111/echo.14172

**Published:** 2018-10-18

**Authors:** Yan Wang, Yinghou Jiang, Hongning Wu, Runfeng Wang, Ying Wang, Cheng Du

**Affiliations:** ^1^ Intensive Care Unit Nanjing Chest Hospital Nanjing China; ^2^ Ultrasonic Department Nanjing Chest Hospital Nanjing China

**Keywords:** inferior vena cava distensibility index, stroke volume variationinnuid, ultrasound, volume responsiveness

## Abstract

**Aim:**

First, the inferior vena cava dilatation index (DIVC) was measured by ultrasound, and then the reliability of DIVC as an indicator to predict volume responsiveness in patients undergoing mechanical ventilation after pneumonectomy was evaluated.

**Methods:**

Pulse indicator continuous cardiac output (Picco) as gold standard was performed to sedated mechanically ventilated post‐pneumonectomy patients in intensive care unit of Nanjing Thoracic Hospital from August 2014 to December 2016. Meanwhile, ultrasound measurement to inferior vena cava (IVC) diameter at the end inspiration (*D*_max_) and the end of expiration (*D*_min_) was performed. DIVC = (*D*_max_ − *D*_min_)/*D*_min_. Above values were recorded at baseline and then after fluid resuscitation challenge (7 mL/kg hydroxyethyl starch). An increase in cardiac index of more than 15% was used as the standard for fluid responsiveness. Patients were divided into responsive group and non‐responsive group. A receiver operating characteristic (ROC) curve was then used to determine the sensitivity and specificity of DIVC in predicting fluid responsiveness after pneumonectomy.

**Results:**

Eighteen patients were enrolled. 10 patients were divided into responsive group and eight in non‐responsive group. DIVC in responsive group was significantly higher than in non‐responsive group (*P *<* *0.01). By setting DIVC ≥ 15% as a measure of fluid responsiveness, sensitivity was 81.8% and specificity was 85.7%.

**Conclusion:**

DIVC is a reliable indicator of capacity responsiveness in mechanically ventilated post‐pneumonectomy patients.

## 1 INTRODUCTION

Pneumonectomy is a common procedure in thoracic surgery and involves perioperative risks of cardiac and pulmonary dysfunction associated with significant mortality.^1^ Perioperative mismanagement of fluid resuscitation is an important factor contributing to poor outcomes.^2^

**Figure 1:**
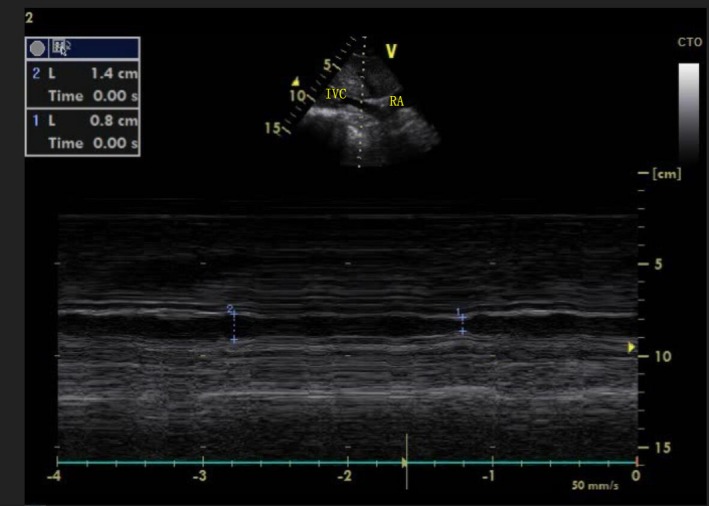
Measuring method of DIVC

In recent years, ultrasound has been widely reported as a modality for predicting fluid responsiveness through measurement of variation in the diameter of the inferior vena cava diameter in patients with sepsis,^3^ subarachnoid hemorrhage,^4^ and trauma.^5^ This study was designed to investigate the reliability of inferior vena cava diameter variation in predicting fluid responsiveness in post‐pneumonectomy patients (Figure 1).

## 2 MATERIALS AND METHODS

### 2.1 Research subjects

Post‐pneumonectomy mechanically ventilated patients requiring fluid resuscitation in intensive care unit of Nanjing Thoracic Hospital from August 2014 to December 2016.

#### 2.1.1 Inclusion criteria

At least one of the following criteria indicative of insufficient perfusion must be met for reperfusion therapy: (a) heart rate > 100/min; (b) systolic blood pressure < 90 mm Hg (or a drop of mean arteriole pressure [MAP] > 30%); (c) urine output < 0.5 mL/kg/h for more than 2 h, or skin tenting.

#### 2.1.2 Exclusion criteria

(a) refusal to sign informed consent; (b) need for protective pulmonary ventilation; (c) contraindicating fluid challenge, such as cardiac insufficiency; (d) inability to obtain satisfactory inferior vena cava image by an experienced ultrasonographer.

### 2.2 Research methods

After adequate sedation, spontaneous breathing was interrupted. The ventilator was adjusted to the A/C mode with tidal volume of 8–12 mL/kg, respiratory rate of 12–20 breaths/min, and positive end‐expiratory pressure (PEEP) of 5–10 mm Hg. An echocardiogram was performed to rule out severe cardiac insufficiency, which is a contraindication to rehydration challenge. DIVC was assessed through ultrasound. Measurement of the diameter of the inferior vena cava was performed through standard ultrasonic techniques. The M‐mode ultrasound probe was positioned longitudinally along the xiphoid process when the patient was supine. The diameter of the inferior vena cava was measured at 2 cm close to the entrance of the right atrium. Ultrasound images were taken at the end of inspiration (*D*_max_) and at the end expiration (*D*_min_) to calculate DIVC. In patients who underwent pneumonectomy, the lack of lung tissue on one side of the thoracic cavity produced a gas‐containing cavity. In a small subset of patients, clear images of the IVC were difficult to obtain from the xiphoid approach. For this reason, these patients underwent ultrasound through a right abdominal flank approach to obtain cross sections of the IVC. Ultrasound measurements to the IVC short diameter, IVC long diameter, and the IVC inner diameter deformation index at various points of the respiratory cycle were closely correlated on the subxiphoid and the right flank. As a result, these two approaches are interchangeable.^6^ We used PICCO to measure the CI prior to fluid resuscitation challenge followed by volume challenge with 6% hydroxyethyl starch (130/0.4) 7 mL/kg (ideal body weight). A constant and rapid infusion was given in 30 minutes followed by a repeated measurement of the CI. The change in CI (∆CI) was then calculated.

Based on prior research and literature,^7^ ∆CI ≥ 15% is defined as fluid responsive. Based on this criterion, we divided our patients into fluid responsive group(R) and fluid non‐responsive group (NR).

Using the SPSS 13.0 (SPSS Inc., Chicago, IL, USA) software package, measurement data were expressed as mean ± standard deviation (X ± S). The paired *t* test was used to compare the IVC diameter as well as the data before and after volume expansion. Comparisons of the measurements between groups were performed using independent sample *T* test. Qualitative measurements were compared using the chi‐squared test. Linear correlation evaluated using the Pearson correlation coefficient. ROC comparison was performed using the Hanley‐McNeil test. The Youden index was used to determining diagnostic thresholds, including sensitivity, specificity, positive predictive value, and negative predictive value.

## 3 RESULTS

1. There were 24 post‐pneumonectomy mechanically ventilated patients in intensive care unit of Nanjing Thoracic Hospital from August 2014 to December 2016. One patient was excluded by pre‐operation examination for bad cardiac function. Four patients were excluded because of having no satisfied images. One patient was excluded because of needing protective pulmonary ventilation. Finally, 18 patients (11 male, 7 female) were enrolled. There were no statistically significant differences in clinical characteristics (such as age and gender) between groups (*P* > 0.05, Table 1).
  **Table 1:** Clinical features

	Group R	Group NR
Gender (male)	5/10	5/8
Age (y)	53.6 ± 5.6	54.8 ± 6.9
BMI (kg/m^2^)	1.95 ± 0.2	1.93 ± 0.2
Tidal volume (mL)	416.3 ± 13.6	412 ± 16.6

BMI = body mass index; PEEP = positive end‐expiratory pressure.
2. Hemodynamics measurement: Patients studied Picco to get CI before and after fluid challenge. △CI > 15% was considered as responsive. According to this index, patients were divided into responsive group(R group) and non‐responsive group (NR group), R group had 10 patients and NR group had 8 patients. There are no significant differences in clinical characteristics (such as age, gender, body mass index, and tidal volume) and hemodynamic information, such as SV, HR, CI, MAP, or central venous pressure (CVP) between the two groups (*P* > 0.05, Tables 1, 2).
  **Table 2:** Hemodynamic index before testing (X ± S)

Hemodynamics index	Group R	Group NR
SVI	56.4 ± 8.8	66.1 ± 15.8
HR	98.6 ± 12.2	93.4 ± 10.3
CI	6.0 ± 0.6	6.7 ± 1.0
MAP	76.5 ± 9.4	79.9 ± 8.7
CVP	12.4 ± 2.0	13.3 ± 2.8

CI = cardiac index; CVP = central venous pressure; HR = heart rate; MAP = mean arteriole pressure; SVI = stroke volume index.
3. Receiver operating characteristic analysis: IVC diameter variation (DIVC) was evaluated using the ROC to evaluate the fluid responsiveness of mechanically ventilated post‐pneumonectomy patients. The area under the ROC curve (AUC) was 0.86. When DIVC ≥ 15% was considered fluid responsive, the sensitivity was 81.8% and specificity was 85.7% (Table 3; *P* = 0.01).
  **Table 3:** Results of receiver operating characteristic analysis for predicting fluid responsiveness with DIVC

Index	Area under the curve	Standard error	Sensitivity	Specificity	Diagnostic value	*P*
DIVC	0.864	0.092	81.8	85.7	0.15	0.011

## 4 DISCUSSION

Pneumonectomy is defined as the complete unilateral lung resection. It is primarily indicated for the treatment of lung cancer and destructive lung disorders. Due to reduced pulmonary vasculature and increased pulmonary circulatory pressure, patients are prone to cardiac insufficiency, pulmonary edema, as well as other complications during the perioperative period. Insufficient perioperative hydration is one of the important causes of postoperative morbidity and mortality.^8^ Inadequate resuscitation may lead to complications caused by insufficient perfusion of the brain, heart, kidneys, and other organs. Perioperative fluid volume management is paramount.

The goal of capacity therapy is to increase the cardiac output. Ultimately, the benefits include declining heart rate, normotension, increased urine volume, and circulatory improvement, also known as fluid responsiveness.

Traditional capacity‐monitoring tools such as CVP and pulmonary artery wedge pressure (PAWP) provide indices of preload pressure.^9^, ^10^ Intrathoracic blood volume index (ITBVI) and global end diastolic volume index (GEDVI) do not predict fluid responsiveness.^11^ This study shows no significant correlation between CVP, ITBVI, GEDVI, and CI (Table 4). This is because fluid responsiveness depends largely on whether the left and right ventricles are consistent with the ascending part of the Frank‐Starling curve and is poorly predicted by the cardiac preload.^12^

**Table 4:** Results of receiver operating characteristic analysis for predicting fluid responsiveness with central venous pressure (CVP), global end diastolic volume index (GEDVI) and intrathoracic blood volume index (ITBVI)

Index	Area under the curve	Standard error	Sensitivity	Specificity	Diagnostic value	*P*
CVP	0.604	0.170	33.33	100	11	0.540
GEDVI	0.626	0.128	88.9	45.5	517.0	0.342
ITBVI	0.535	0.134	100	27.3	607.5	0.790

Currently, common methods in monitoring fluid responsiveness include rapid rehydration challenge and passive leg raise (PLR). To measure indicators of cardiopulmonary interaction such as systolic pressure variation (SPV), pulse pressure variation (PPV), and stroke volume variation (SVV) by Picco.^13^ These methods may increase the risk of the fluid overload and may be limited by accuracy or range of indications. A simple and reliable method to predict and evaluate fluid responsiveness is needed.

In recent years, the advancement of ultrasound technology led to its increased role in critical care. It is generally accepted that the IVC is a reliable index suitable for evaluating hemodynamic states.^14^ In patients with low blood volume, the IVC diameter is significantly smaller in normal patients.^15^ In patients with fluid overload, the IVC is dilated and fixed. The IVC variation index has been confirmed to predict the fluid responsiveness in critically ill patients.^3^, ^4^, ^5^, ^16^ This study is the first to apply IVC diameter variation in pneumonectomy patients. Our research confirms that DIVC can accurately predict the fluid responsiveness in mechanically ventilated pneumonectomy patients. With DIVC ≥ 18% being fluid responsive, the sensitivity and specificity in predicting fluid responsiveness are 81.8% and 85.7%, respectively. IVC ultrasound is more accessible and simpler to interpret than methods commonly used for the assessment of fluid responsiveness (IVC size and DIVC can be completed within 3 minutes by ultrasound‐trained ICU physicians). IVC ultrasound is noninvasive, does not involve radiation, and can be easily repeated. Cardiac ultrasonography can also assess ejection fraction, right atrial pressure, right ventricular end‐diastolic volume, left ventricular end‐diastolic volume, and velocity time integral of aortic valve (VTI), which are also predictive of fluid responsiveness.^13^ Future combinatorial analysis of these additional measurements may further increase the predictive accuracy of DIVC.

This study has some limitations. First, because IVC diameter and DIVC depends on the pressure surrounding the IVC (intrathoracic and intra‐abdominal pressures) and the right atrial pressures, IVC diameter and DIVC may not be predicted accurately in patients with right cardiac insufficiency, severe tricuspid stenosis, or abdominal compartment syndrome.^4^ Patients with pneumonectomy have lost nearly half of their pulmonary vascular beds and this postoperative increase in pulmonary circulation pressure may cause right cardiac insufficiency, which may further impact the accuracy of DIVC, necessitating further research. Second, the current work included only a limited number of cases. A larger study is needed to further evaluate the accuracy of the diagnostic threshold for DIVC in mechanically ventilated pneumonectomy patients. Third, ultrasound assessment of IVC may be limited in certain pneumonectomy patients for various reasons (ie, obesity, side of surgery, pneumothorax).

In conclusion, ultrasound is a quick, safe, and reliable method of assessing variations in IVC diameter for the prediction of fluid responsiveness in postoperative pneumonectomy patients.
